# The surface properties of *Shewanella putrefaciens* 200 and *S. oneidensis* MR-1: the effect of pH and terminal electron acceptors

**DOI:** 10.1186/1467-4866-14-3

**Published:** 2013-04-08

**Authors:** Yoko Furukawa, Jason R Dale

**Affiliations:** 1 Naval Research Laboratory, Seafloor Sciences Branch, Stennis Space Center, MS, 39529, USA

**Keywords:** Ohshima, *Shewanella*, DLVO, Soft particle, Electrophoretic mobility, Colloid, Floc, Flocculation, Aerobic, Anaerobic, TMAO

## Abstract

**Background:**

We investigated the surface characteristics of two strains of *Shewanella sp*., *S*. *oneidensis* MR-1 and *S*. *putrefaciens* 200, that were grown under aerobic conditions as well as under anaerobic conditions with trimethylamine oxide (TMAO) as the electron acceptor. The investigation focused on the experimental determination of electrophoretic mobility (EPM) under a range of pH and ionic strength, as well as by subsequent modeling in which *Shewanella* cells were considered to be soft particles with water- and ion-permeable outermost layers.

**Results:**

The soft layer of *p*200 is significantly more highly charged (i.e., more negative) than that of MR-1. The effect of electron acceptor on the soft particle characteristics of *Shewanella sp*. is complex. The fixed charge density, which is a measure of the deionized and deprotonated functional groups in the soft layer polymers, is slightly greater (i.e., more negative) for aerobically grown *p*200 than for *p*200 grown with TMAO. On the other hand, the fixed charge density of aerobically grown MR1 is slightly less than that of *p*200 grown with TMAO. The effect of pH on the soft particle characteristics is also complex, and does not exhibit a clear pH-dependent trend.

**Conclusions:**

The *Shewanella* surface characteristics were attributed to the nature of the outermost soft layer, the extracellular polymeric substances (EPS) in case of *p*200 and lypopolysaccharides (LPS) in case of MR1 which generally lacks EPS. The growth conditions (i.e., aerobic vs. anaerobic TMAO) have an influence on the soft layer characteristics of *Shewanella sp*. cells. Meanwhile, the clear pH dependency of the mechanical and morphological characteristics of EPS and LPS layers, observed in previous studies through atomic force microscopy, adhesion tests and spectroscopies, cannot be corroborated by the electrohydrodynamics-based soft particle characteristics which does not exhibited a clear pH dependency in this study. While the electrohydrodynamics-based soft-particle model is a useful tool in understanding bacteria’s surface properties, it needs to be supplemented with other characterization methods and models (e.g., chemical and micromechanical) in order to comprehensively address all of the surface-related characteristics important in environmental and other aqueous processes.

## Background

The surface electrochemical structures of microbial cells in aqueous suspension affect a large variety of environmentally significant interfacial processes, such as biofouling, corrosion, colloid flocculation, and sorption of contaminants. For example, the biofouling of reverse osmosis membrane can be predicted by the surface electrochemical properties of the microbes [1]. The transport behavior of *Escherichia coli* isolates through porous media depends on the bacteria’s surface electrochemical and physical properties [2]. The contaminant sorption on bacteria occurs primarily on the cell walls and extracellular polymeric substances (EPS) whose electrochemical and molecular properties in turn determine the sorption mechanisms and magnitudes [3,4]. In addition, the interaction between microbial cells, EPS and surrounding environment are important in coastal sediment stabilization, nutrient scavenging, biofilm stabilization due to gene transfer, and carbon cycling [5-14].

Electrokinetic measurements (i.e., electrophoresis) are often used to characterize the surface electrochemical structure of colloidal particles in aqueous suspension. For colloidal particles with impermeable surfaces, such as latex and mineral particles, electrophoretic mobility (EPM) data can readily yield a quantitative description of the surface electrochemical structure using the electric double layer (EDL) model framework [15]. In the EDL model, a negatively (or positively) charged mineral surface is surrounded by a layer of electrostatically attracted cations (or anions) as the counter ions. Further, this inner layer is surrounded by a so-called diffuse layer, which differs from the electrically neutral bulk aqueous solution in that it is locally enriched with cations (or anions) while depleted with anions (or cations) due to the long-range electrostatic interaction with the mineral surface. For rigid particles, careful analyses of EPM data can yield the electrical potential at the surface (or zeta potential at the slip plane as its empirical proxy), as well as the attenuation of the potential within EDL. A quantitative understanding of the surface electrochemical structure allows a quantitative and predictive understanding of flocculation, transport, and sorptive properties of the colloidal particles in aqueous suspension by employing the DLVO theory (Derjaguin and Landau [16], Verwey and Overbeek [17]) [18-20].

**Figure 1:**
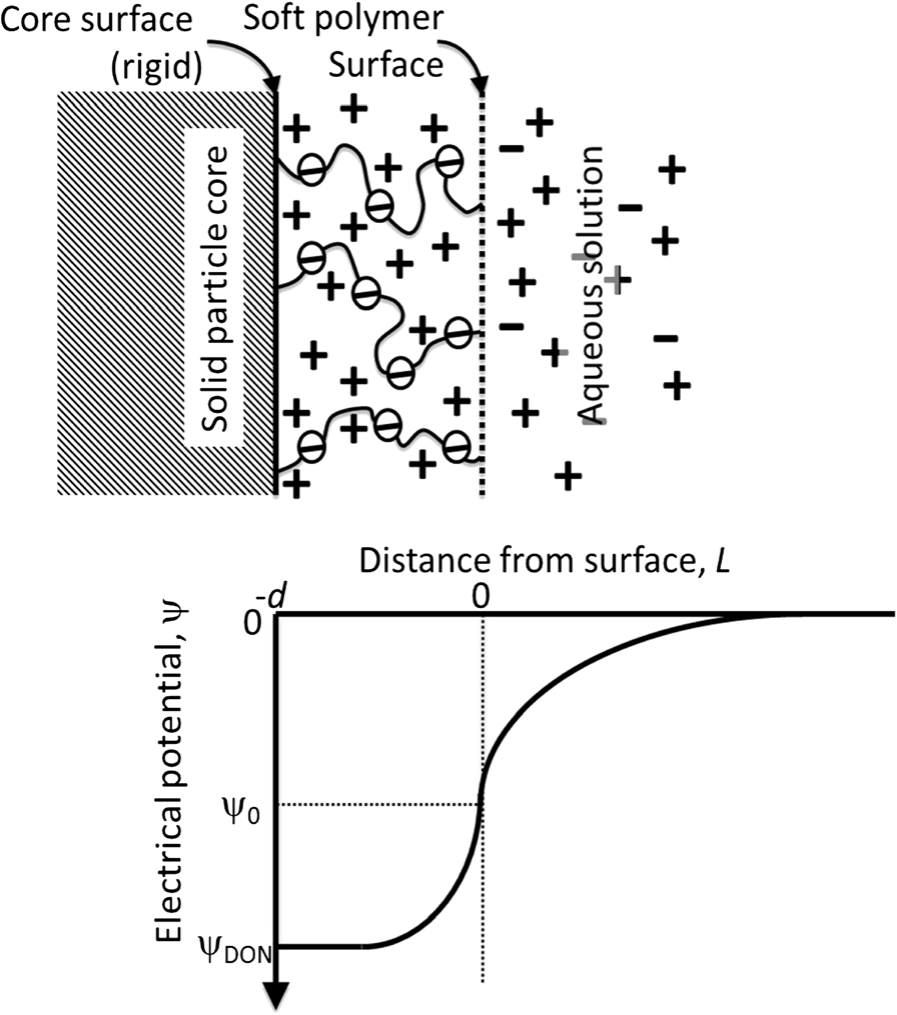
**Ion and charge distribution and electrical potential attenuation across a surface soft layer of thickness *d *containing negatively charged (deprotonated) functional groups.** The Donnan potential, ψ_DON_, represents the potential at the rigid surface of the particle (*L* = −*d*), whereas the surface potential, ψ_0_, is the potential at the soft particle surface (*L* = 0). The fixed charge density, ρ_fix_, represents the charge density within the soft polymer layer that arises from the deprotonated functional groups of the polymer segment. The softness parameter, 1/λ, is a relative measure of the ease of water flow within the soft later. After Ohshima [22].

However, soft particles, i.e., particles with water- and ion-permeable surface layers (i.e., “soft layers”), do not allow such a straight-forward interpretation of the electrokinetic data [21,22]. The bulk behavior of suspended soft particles, such as flocculation, cannot always be predicted from the electrokinetic data alone [23]. The EPM of a soft particle depends on the attenuation of electrical potential in the vicinity of the surface, which in turn depends not only on the properties of host aqueous solution but also on the water and ion permeability of the soft layer. Ohshima has developed a model in which experimentally measured EPM can be correlated to key parameters that describe the surface electrochemical structures of soft particles, i.e., Donnan potential, surface potential, fixed charge density and softness parameter (Figure 1) [22,24].

Microbial cells may be considered using the soft particle model. The cell wall of gram-negative bacteria consists of an outer membrane containing lipopolysaccharides (LPS), as well as a gel-like periplasm with a thin peptidoglycan layer. These outer membrane components are permeable to water molecules and ions. In addition, the extracellular polymeric substances (EPS), often produced by microorganisms, are also permeable. Recently, the EPM data of *Shewanella sp*. and other bacteria have been evaluated using Ohshima’s soft particle model [25-28]. The effect of ionic strength on the soft particle properties of *Shewanella sp*. has been found to be quantitatively significant in *S*. *putrefaciens* CN32, which is surrounded by a thin, charged envelope, while it was found to be less pronounced in *S*. *oneidensis* MR-4, which is surrounded by a thick gel-like layer [29].

*Shewanella sp*. has been extensively studied as a model microorganism due to its ubiquitous presence in a wide range of natural and engineered environments, respiratory versatility and ease of genetic manipulation [30-32]. Its ability to conduct dissimilatory metal reduction has been exploited for potential applications in the bioremediation of metal and organic contaminants [33]. A better understanding of the *Shewanella sp*. surfaces in terms of their electrochemical structures and consequential flocculation, transport and sorptive properties would enable: (i) a further interpretation of the existing *Shewanella sp*. knowledge base; and (ii) better guiding of the future designs for bioremediation applications utilizing *Shewanella sp*. and other similar microorganisms.

Previous studies have found that pH has a quantitatively significant influence on the mechanical and morphological properties of the biopolymers that surround *Shewanella sp*. cells [34-36]. The cell surface soft particle properties have been found to differ between cells grown with TMAO and with fumarate/nitrate as electron acceptors [26]. The objective of this study is to investigate the effect of pH and electron acceptors (O_2_ vs. trimethylamine oxide (TMAO)) on the soft particle properties of EPS-poor and EPS-rich gram negative bacteria, *Shewanella oneidensis* MR-1 and *S*. *putrefaciens* 200, respectively.

## Experimental and modeling

### *Shewanella* preparation

*Shewanella oneidensis* MR-1 (ATCC 7005500) (herein referred to as MR1) and *Shewanella putrefaciens* 200 (ATCC 51753) (herein referred to as p200) stock cultures were maintained in Luria-Bertani medium with 20% glycerol at −80°C and were routinely grown in LB medium at 30°C for 15 h on a rotary shaker (150 r.p.m.). Water for all experiments was supplied from a Millipore (Direct-Q 5) ultrapure water system. EPS was produced in cultures containing 1 L modified M1 medium containing 3.0 mM PIPES, 7.50 mM NaOH, 28.04 mM NH_4_Cl, 1.34 mM KCl, 4.35 mM NaH_2_PO_4_ and 0.70 mM CaCl_2_ supplemented with trace amounts of minerals, vitamins and amino acids [37,38]. Thirty mM Na-lactate was added as electron donor and 30 mM trimethylamine oxide (TMAO) was added as electron acceptor in anaerobic cultures. Air or N_2_ was bubbled through the medium to maintain aerobic or anaerobic growth conditions, respectively. Cultures were inoculated (OD_600_ = 0.1) and grown to late exponential phase (24 h). Dague and colleagues have shown that the EPM of *Shewanella* cells does not vary appreciably between cells harvested at different growth periods (midexponential vs. pseudostationary) [25].

### Electrophoretic mobility (EPM)

Laser Doppler velocimetry (LDV) analysis was used to determine the EPM of MR1 grown under either oxygen or TMAO as electron donors (referred as MR1O2 and MR1TMAO) and p200 grown under either oxygen or TMAO as electron donors (referred as p200O2 and p200TMAO) under a range of ionic strength (20 ≲ *I* ≲ 200 mol m^-3^) and pH (2 ≲ pH ≲ 12). The analysis was conducted using the Malvern Zetasizer nano-ZS at 25°C.

Approximately 10 ml of each bacteria suspension sample, with prescribed adjustments to ionic strength using KCl, a monovalent electrolyte, was loaded into a sample reservoir, from which a small aliquot (~ 1 mL) was introduced to the LDV capillary chamber with embedded electrodes. Suspended cells, whose surface was charged either positively or negatively depending on the pH, moved towards the electrode of the opposite charge when the potential was applied, and their average velocity was measured. By knowing the physical properties of the suspension medium, the velocity was converted to EPM [39]. The LDV techniques have been previously used to characterize the EPM of various naturally-derived colloids including bacterial cells and EPS [40].

For each series of analysis, the ionic strength was held approximately constant whereas pH was varied by titrating the reservoir sample with the addition of 0.1 N HCl or 0.1 N NaOH. After each pH adjustment monitored by a combination pH electrode, the reservoir suspension was homogenized with a magnetic stirrer before a ~ 1 mL aliquot was introduced to the LDV capillary chamber. After the analysis, the aliquot was returned back to the reservoir, homogenized with the rest of the reservoir sample, and a small fraction was introduced to the capillary chamber again for an additional analysis. The analysis was conducted three times for each pH value. Once in the capillary chamber, the conductivity of the suspension solution was determined along with the EPM. The conductivity was later converted to ionic strength. The conductivity (and thus ionic strength) varied slightly during each titration series due to the NaOH or HCl addition.

### Ohshima soft particle model and optimization

#### Ohshima model

Ohshima has shown that the EPM of soft particles, *μ*, can be related to four key parameters, Donnan potential, ψ_DON_, surface potential, ψ_0_, fixed charge density, ρ_fix_, and electrophoretic softness, λ, as follows [22]:

(1) $$\mu =\frac{\varepsilon_{0}\varepsilon_{r}}{\eta }\frac{\frac{\psi_{0}}{k_{m}}+\frac{\psi_{\text{DON}}}{\lambda }}{\frac{1}{k_{m}}+\frac{1}{\lambda }}+\frac{\text{ρfix}}{\eta \lambda^{2}}$$

where fixed charge density, ρ_fix_, is defined by the number concentration (*N*) and valence (*Z*) of the dissociated functional groups in the soft layer as well as the elementary electric charge (*e*):

(2) ρfix=NZe

and electrophoretic softness, 1/λ, of the soft layer is defined by the viscosity of the aqueous medium (η) and the soft layer’s friction coefficient (ω):

(3) $$\frac{1}{\lambda }=\sqrt{\frac{\eta }{\omega }}$$

In reality, the electrophoretic softness can be considered as the relative measure of the ease of flow penetration into the soft layer [35]. In equation (1), ε_0_, ε_r_, and η are the permittivity of vacuum, relative permittivity, and viscosity of the aqueous medium, respectively. Further, Ohshima has shown that the rest of the parameters can be expressed as follows [22]:

(4) $$\psi_{\mathrm{DΟΝ}}=\frac{\text{kT}}{\mathrm{ze}}\ln\left(\frac{\text{ρ}\text{fix}}{2\text{zen}\text{∞}}+\sqrt{{\left(\frac{\text{ρ}\text{fix}}{2\text{zen}\text{∞}}\right)}^{2}+1}\right)$$

(5) $$\psi_{0}=\psi_{\text{DON}}+\frac{\mathrm{kTn\infty}}{\text{ρ}\text{fix}}\left(1-\sqrt{{\left(\frac{\text{ρ}\text{fix}}{2\text{zen}\text{∞}}\right)}^{2}+1}\right)$$

(6) $$km=k{\left(1+{\left(\frac{\text{ρfix}}{2\text{zen∞}}\right)}^{2}\right)}^{1/4}$$

where *k* is the Boltzmann constant, *T* is the absolute temperature, *z* and *n*^∞^ are the valence and number concentration of the dissolved electrolyte in the bulk aqueous solution, and κ is the reciprocal of the Debye length $\left(k^{-1}=\sqrt{\frac{\varepsilon_{r}\varepsilon_{0}\text{kT}}{2e^{2}\text{zn∞}}}\right)$, in monovalent electrolyte solution.

Ohshima’s soft particle model assumes that the soft layer is homogeneous in terms of the permeability and charge distribution. However, soft layers of natural bacteria may be considered as diffuse [29]. Recent studies have shown that a more rigorous treatment of the charge and permeability distributions within the soft layers using a step function or numerical solution can successfully depict the heterogeneity [21,41]. However, Ohshima [41] has shown that the effect of inhomogeneity within soft layer becomes insignificant at moderate to high ionic strength. Recent soft-particle analyses of *Shewanella sp*. show that errors due to the homogeneity assumption are small when *I* ≳ 0.02 (M) [21]. A similar result was also observed with *Pseudomonas sp*[28]. Consequently, our study utilized EPM data that were obtained at 0.02 ≲ *I* (M).

Ohshima’s model also assumes that the particle size is much greater than the Debye length and thus the particle surface is approximated to be planer [24,41]. The Debye length at *I* ≥ 0.02 (M) is κ^-1^ ≤ 2.1 (nm) while a typical size of *S*. *sp*. cells is several hundred nanometers in width and a few thousand nanometers in length (e.g., [42]). Consequently, the planer assumption is valid when the EPM data obtained at 0.02 ≲ *I* (M) are considered.

#### Optimization

By substituting Equations (4) – (6) in (1), it is evident that the EPM can be expressed with just two of the key parameters, fixed charge density, ρ_fix_, and electrophoretic softness, λ^-1^, under given ionic strength values (which is linearly related to n^∞^).

An optimization scheme (i.e., the *lsqnonlin* routine on Matlab®) was used with the EPM data obtained at 0.02 ≲ *I* ≲ 0.15 (M) and at pH values within discrete ranges in order to determine the best ρ_fix_ and λ^-1^ values for each of the four systems at given pH ranges. In order to eliminate false results due to local minima, the optimization routine was run 100 times using different, randomly generated initial guess values for ρ_fix_ and λ^-1^.

## Results and discussion

### LDV results

**Figure 2:**
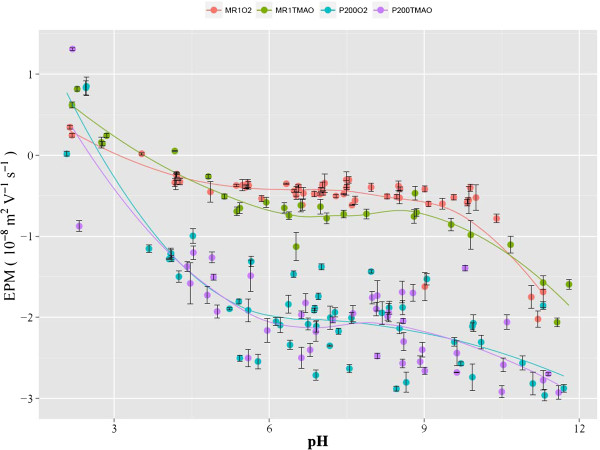
**Experimentally determined EPMs are plotted against pH.** The average from each triplicate measurement is reported, along with the standard deviation shown as error bars. Results from all ionic strength values are shown together on this figure.

**Figure 3:**
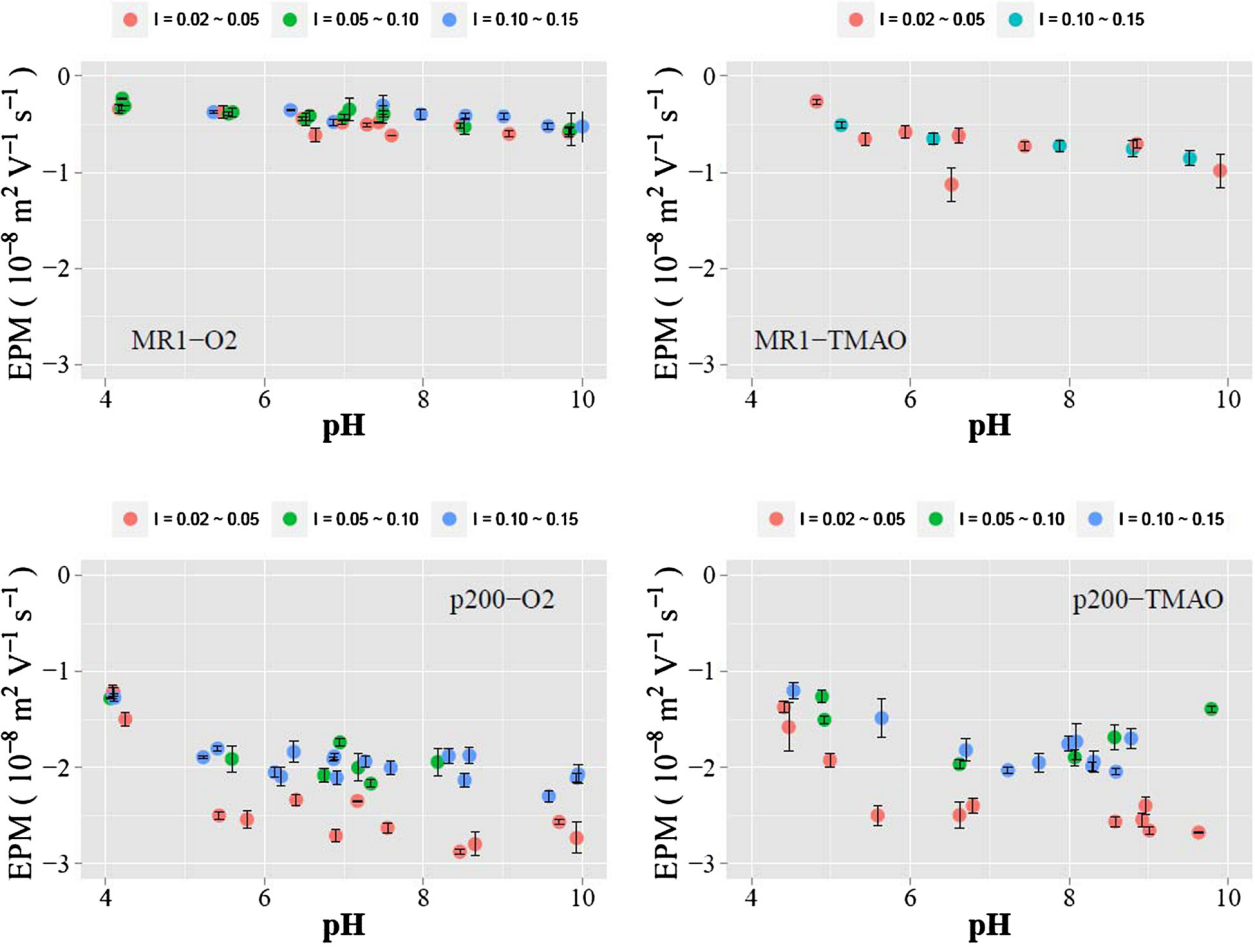
**Experimentally determined EPMs are plotted against pH.** Results from three discrete ionic strength ranges are shown in each panel using different colors.

**Figure 4:**
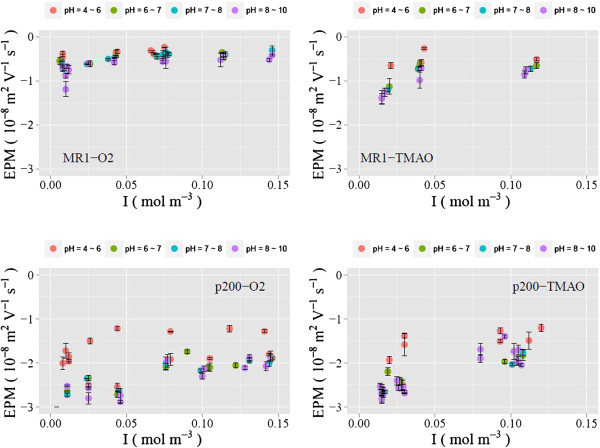
**Experimentally determined EPMs are plotted against ionic strength.** Results from four discrete pH ranges are shown in each panel using different colors.

The experimentally determined EPM values are plotted in Figures 2 and 3 (pH vs. EPM) and Figure 4 (ionic strength vs. EPM). The results indicate that the EPM is greater in magnitude (i.e., more negative) for the EPS-rich *p*200 than for EPS-poor MR1 regardless of the electron acceptors. This is in contrast to a previous study in which the cell walls had greater charge density than the EPS [43]. However, another study revealed that the difference between the relative charge between EPS and cell walls is species- and strain-dependent [44]. Consequently, the results obtained here are specific to *p*200 and MR1, and are not applicable in comparing the cell walls and EPS of other microorganisms.

The isoelectric point (iep) for each system was determined to be iep_MR1O2_ = 3.2, iep_MR1TMAO_ = 3.8, iep_p200O2_ = 2.8, and iep_p200TMAO_ = 2.6. These iep estimates assumed that the specific ion adsorption was negligible in these systems and thus iep is independent of ionic strength.

A recent study has shown that the MR1 surfaces are associated with a patchy presence of EPS while the *p*200 surface has a more extensive EPS coverage [26,45]. The dependence of EPM on ionic strength is more pronounced for the EPS-rich *p*200 than for EPS-poor MR1 at all pH values examined (e.g., Figure 4). This is in contrast to a previous study in which the EPM of gram-positive *Bacillus licheniformis* S-86 exhibited an increased ionic strength dependency when its EPS was removed [40]. The reason for this contrast is unknown. However, we could speculate that, in the absence of EPS, the LPS layer, which is unique to gram-positive bacteria, has significantly different electrohydrodynamic properties compared to the peptidoglycan, which is the outermost layer of gram-positive bacterial cells.

### Fixed charge density and electrophoretic softness

**Figure 5:**
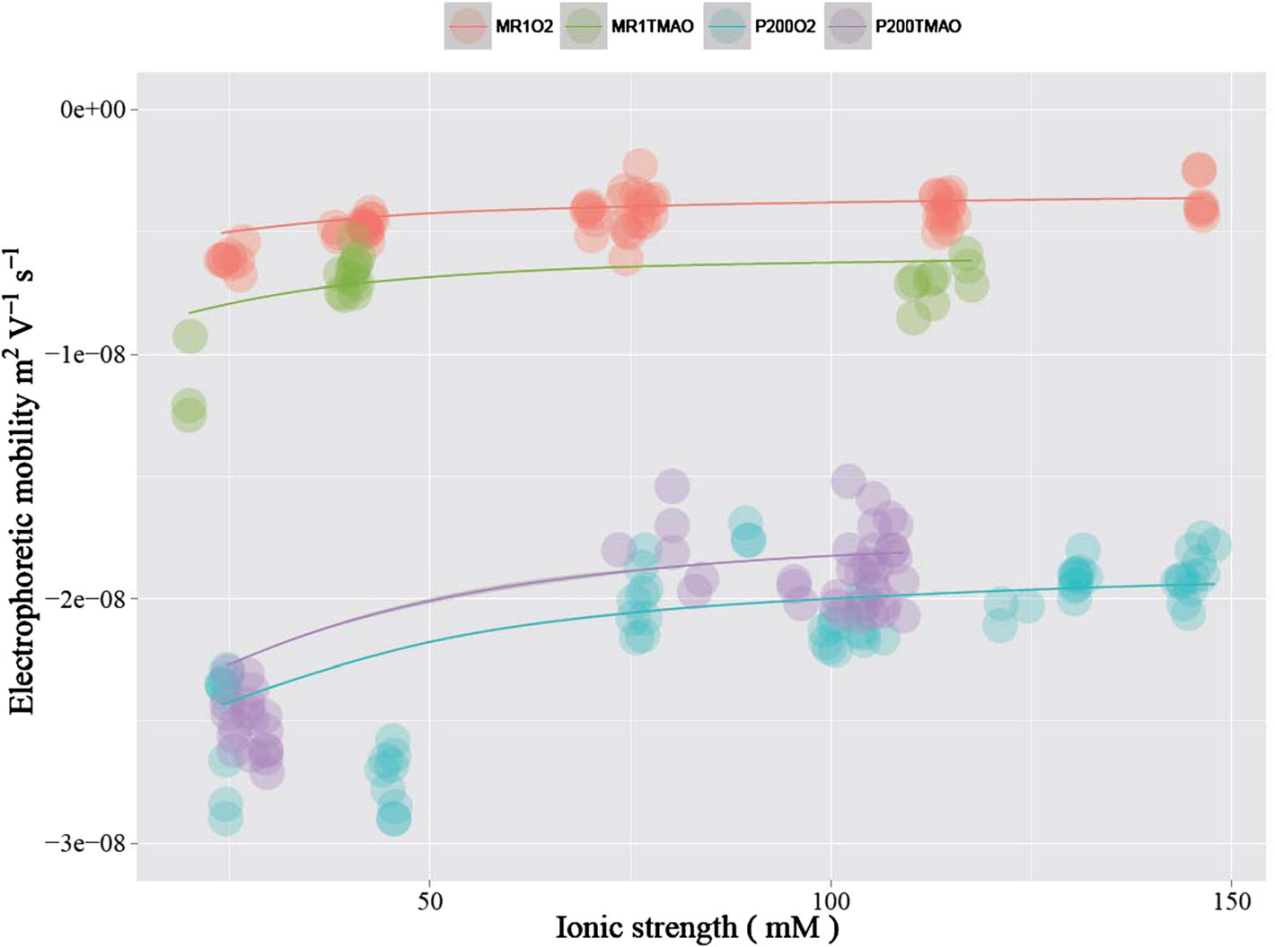
**Experimentally measured EPMs for 6 ≲ pH ≲ 9 are plotted as a function of ionic strength.** In addition, the optimized EPM function (Equation (1) calculated using the optimized λ^-1^ and ρ value pairs) are shown as lines. The optimized λ^-1^ and ρ value pairs used to calculate the best-fit EPM function lines are reported in Table 1.

The measured EPM data were fit to Ohshima’s soft particle model (Equation (1)) with λ^−1^ and ρ_fix_ as the parameters to be optimized. The fitting was conducted using the data points from 0.02 ≲ *I* ≲ 0.15 (M) and discrete pH value ranges in conjunction with the *lsqnonlin* routine in Matlab®. It should be noted that each of the triplicate measurements at a given pH and ionic strength was treated as a separate data point for the model fitting. Table 1 reports the optimized λ^−1^ and ρ_fix_ value pairs for each sample series at each discrete pH ranges. Figure 5 illustrates an example of the best-fit EPM function (i.e., calculated curve for Equation (1) with the optimized λ^−1^ and ρ_fix_ values) for each sample series obtained from the pH range of 6 ≲ pH ≲ 9.

**Table 1:** **Model-determined λ**^**-1 **^**and ρ**_**fix **_**values**

	**pH**	**λ**^**-1**^**(× 10**^**-9**^ **m)**	**ρ**_**fix**_**(× 10**^**5**^**C m**^**-3**^**)**
**MR1O2**	6 – 9 (Figure 5)	1.7	−9.3
	4 – 5	Not enough data	
**MR1O2**	5 - 6	Not enough data	
	6 - 7	2.3	−6.1
	7 - 8	1.4	--11.6
	8 - 9	2.0	−8.2
	9 - 10	3.2	−4.2
	**pH**	**λ**^**-1**^**(× 10**^**-9**^ **m)**	**ρ**_**fix**_**(× 10**^**5**^**C m**^**-3**^**)**
**MR1TMAO**	6 – 9 (Figure 5)	2.1	−11.0
	4 – 5	Not enough data	
	5 – 6	3.0	−4.6
	6 - 7	1.4	−17.9
	7 - 8	2.0	−13.5
	8 - 9	2.2	−9.7
	9 - 10	2.2	−13.6
	**pH**	**λ**^**-1**^**(× 10**^**-9**^ **m)**	**ρ**_**fix**_**(× 10**^**5**^**C m**^**-3**^**)**
**p200O2**	6 – 9 (Figure 5)	2.4	−28.0
	4 – 5	3.5	−8.2
	5 – 6	2.1	−33.1
	6 – 7	2.6	−23.2
	7 - 8	2.5	−26.0
	8 - 9	1.8	−46.4
	9 - 10	3.0	−19.9
	**pH**	**λ**^**-1**^**(× 10**^**-9**^ **m)**	**ρ**_**fix**_**(× 10**^**5**^**C m**^**-3**^**)**
**p200TMAO**	6 – 9 (Figure 5)	2.1	−31.8
	4 – 5	2.5	−16.3
	5 – 6	2.3	−22.8
	6 - 7	2.3	−28.0
	7 - 8	2.6	−21.9
	8 - 9	2.2	−30.0
	9 – 10	2.5	−26.2

The pH values below pH 4 were not considered as the EPM measurements in the vicinity of iep (i.e., pH ≈ 3) were unstable. The pH values above pH 10 were not considered because there was a significant EPM data scatter in that pH region which made the regression optimization unreliable. The scatter may be due to the heterogeneity of the physiological response of *Shewanella* species to increased pH [46].

**Figure 6:**
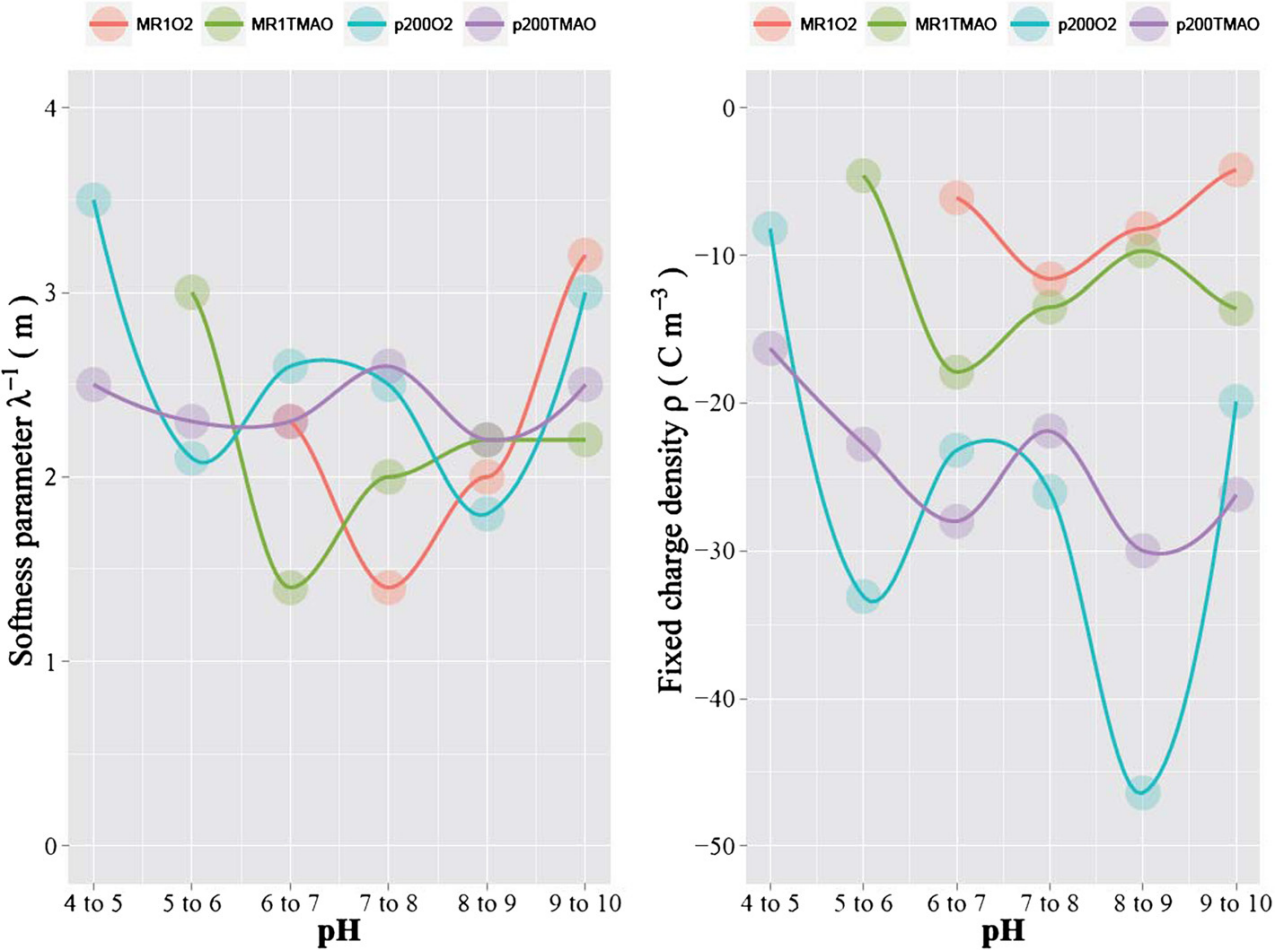
**The softness parameter and fixed charge density values calculated by fitting Ohshima’s soft particle model (Equation (**1**) while adjusting the softness parameter and fixed charge density values to achieve the best fit.** The model calculation was conducted separately for each of the discrete pH ranges. The actual values are shown in Table 1.

Even though both MR1 and *p*200 exhibit EPM data profiles that are typical of soft particles (e.g., Figures 2, 3, 4), they differ in the magnitudes of soft particle characteristics. In our study, the difference in softness parameter between MR1O2, MR1TMAO, P200O2 and P200TMAO were relatively small for the entire pH range investigated (Figure 6). The softness parameter values fell between 1.4 and 3.5 which indicates the presence of a surface layer with a finite permeability value. For comparison, this range is in line with the softness parameter values calculated in the previous studies of various *Shewanella sp*. cultures (e.g., λ^−1^ = 2 – 3.6 (× 10^-9^ m) at pH = 7 for *S*. *oneidensis* MR-4 and CN32 [25,29]; λ^−1^ = 4 (× 10^-9^ m) at pH = 4 – 10 for *S*. *putrefaciens* CIP 80.40 [35] and λ^−1^ = 0.4 – 3 (× 10^-9^ m) at pH = 5.5 – 5.8 for MR1 and p200 grown with TMAO, fumarate and nitrate [26]). On the other hand, our study found that there is a clear difference in the fixed charge density between MR1 and *p*200. The fixed charge density of MR1 was found to vary between −4 and −18 (× 10^5^ C m^-3^) (compared to ρ = ~ − 8 (× 10^5^ C m^-3^) for MR1 grown with fumarate, nitrate or TMAO in a previous study [26]). Meanwhile, it was significantly greater for *p*200 with the values between −8 and −46 (C m^-3^) (compared to ρ = −21 (× 10^5^ C m^-3^) for p200 grown with TMAO and ρ = −43 (× 10^5^ C m^-3^) for p200 grown with fumarate/nitrate in a previous study [26]).

**Figure 7:**
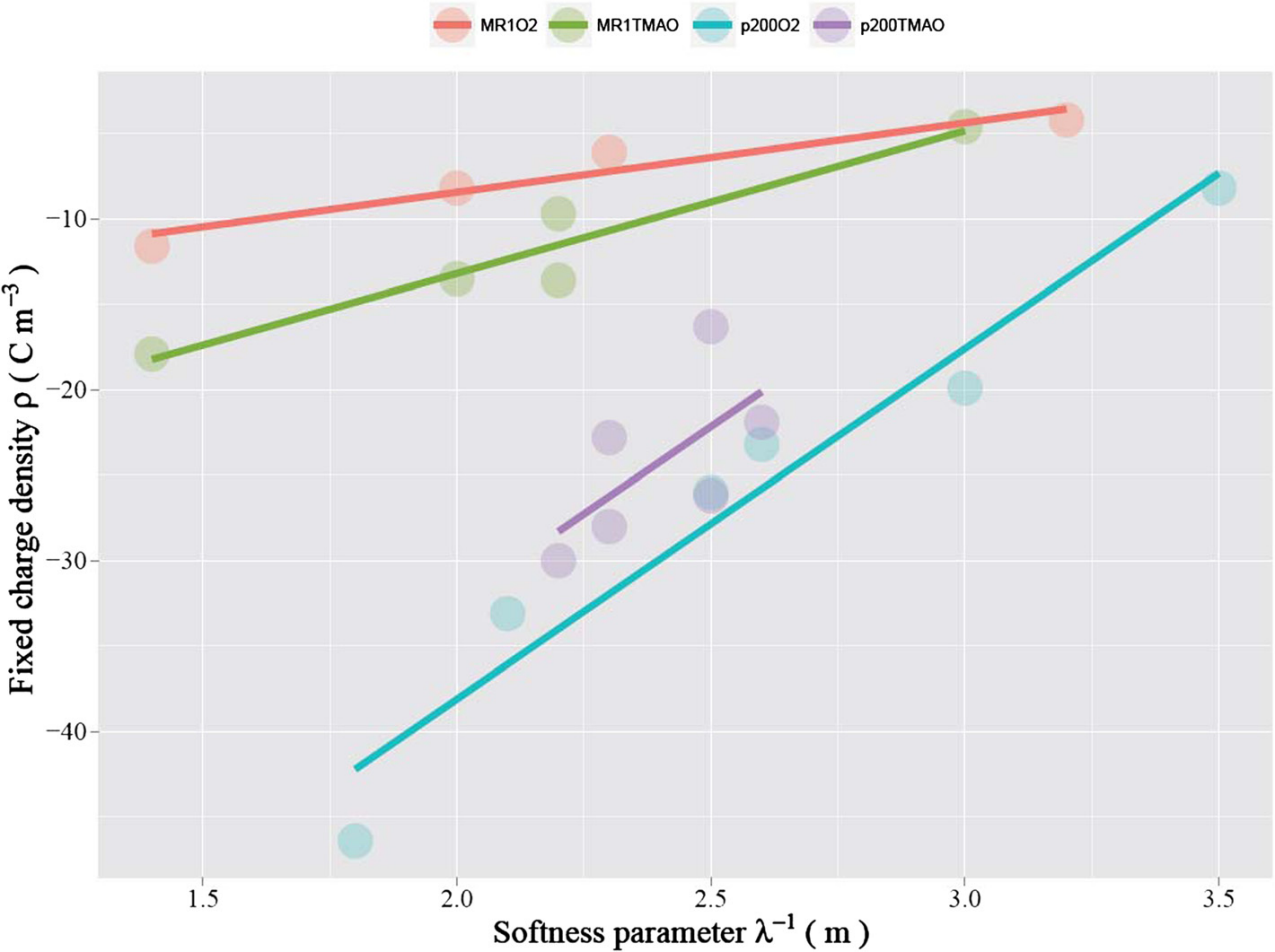
**Relationship between softness parameter and fixed charge density that were determined by fitting Ohshima’s soft particle model (Equation (**1**)) while adjusting the softness parameter and fixed charge density values to achieve the best fit.** The values are shown in Table 1.

The relationship between model-calculated softness parameter and fixed charge density (Figure 7) has clear trends that can be summarized by: (i) the inverse relationship between λ^−1^ and ρ_fix_ (i.e., increased softness parameter is met with less negative fixed charge density); and (ii) more pronounced correlation between λ^−1^ and ρ_fix_ in the EPS-rich *p*200 than in EPS-poor MR1 (i.e., a greater λ^−1^/ρ_fix_ slope for *p*200 than for MR1). The former trend is intuitively apparent. The expansion of the polymer segments leads to more permeable soft layers (i.e., a greater softness parameter) while reducing the number of available ion exchangeable sites per a unit volume (i.e., a decreased fixed charge density). However, it should be noted that there is no clear pH- or electron acceptor-related trend for the polymer expansion and contraction (see below). The latter implies that the materials (polymers) that make up the EPS have more cation exchangeable sites per unit length than the materials that make up the LPS.

The complex effect of different electron acceptors (i.e., oxygen and TMAO) on *Shewanella*’s surface electrohydrodynamic properties can be seen in the relationship between softness and fixed charge density (Figure 7). A recent study has found that both MR1 and *p*200 exhibit significantly enhanced electrophoretic softness when grown with TMAO than with nitrate or fumarate [26]. The same study also found that the fixed charge density of TAMO-grown *p*200 is far less negative than nitrate/fumarate-grown *p*200 [26]. Our study, on the other hand, reveals that the difference between TMAO- and O_2_-grown cells is less straightforward. In our study, TMAO resulted in the decreased (i.e., less negative) fixed charge density over O_2_ for *p*200 while it resulted in the increased (i.e., more negative) fixed charge density over O_2_ for MR1. However, for both cases, the difference is small (Figure 7).

The effect of pH on *Shewanella*’s surface electrohydrodynamic properties was found to be very complex (Figure 6). Generally speaking, the effect of pH on the surface surrounded by biopolymers is two-fold: (i) on one hand, a pH increase causes deprotonation of ionizable functional groups in the biopolymers which would result in increased (i.e., more negative) fixed charge density [40,46]; (ii) on the other hand, the increased pH and functional group deprotonation lead to electrostatic repulsion between the negatively-charged ligands as well as polymer segments, and a consequential volume expansion in the biopolymer volume and decreased (less negative) fixed charge density [35,40]. In our study, the fixed charge density does not have a clear dependency on pH. There may be a slight tendency for more permeable soft layer (i.e., high λ^-1^) at extreme low and high pH values while less permeable soft layer (i.e., low λ^-1^) at circumneutral pH values. It is likely that the effect of pH on the soft particle properties of MR1 and p200 results from a complex interplay between the above (i) and (ii) as well as the physiological and chemical responses of *Shewanella* cells to different pH values.

Studies have shown that the morphological and mechanical properties of the outermost soft layers of bacteria cells, including *Shewanella sp*., change in response to pH changes [36]. An ATR-FTIR spectroscopy study revealed that EPS-poor *S*. *CN32* (may be analogous to MR1 in our study) changes the chemical properties of its LPS functional groups with pH driven by protonation and deprotonation [34]. The LPS and EPS layers of *S*. *putrefaciens* CIP 80.40 (may be analogous to *p*200 in our study) increase their volume and permeability with increasing pH, as investigated by atomic force microscopy and microbial adhesion tests [35]. However, these linear or semi-linear correlation between pH and morphological/mechanical/chemical properties in EPS and LPS layers are not reflected in the soft particle parameters derived from Ohshima’s electrohydrodynamic soft particle theory [24] for our study. The pH dependency observed in our study is, for the most part, variable and nonlinear. This suggests that, while the electrohydrodynamics-based soft-particle model is a useful tool in investigating bacteria’s aqueous aggregation behavior and other behaviors controlled by the surface processes, it may be inadequate in comprehensively addressing the surface characteristics including the chemical and micromechanical characteristics. The comprehensive characterization of the bacterial cell surfaces would require the employment of multiple analytical techniques, including electrohydrodynamic, micromechanical, and chemical techniques.

## Conclusions

The *Shewanella* surface characteristics were attributed to the nature of the outermost soft layer, the extracellular polymeric substances (EPS) in case of the EPS-rich *p*200 and the cell wall lypopolysaccharides (LPS) in case of the EPS-poor MR1. The growth conditions (i.e., aerobic vs. anaerobic TMAO) have an influence on the soft layer characteristics of *Shewanella sp*. cells. Meanwhile, the clear pH dependency of the mechanical and morphological characteristics of EPS and LPS layers, observed in previous studies through atomic force microscopy, adhesion tests and spectroscopies, cannot be corroborated by the electrohydrodynamics-based soft particle characteristics which do not exhibited a clear pH dependency in this study. While the electrohydrodynamics-based soft-particle model is a useful tool in understanding bacteria’s surface properties, it needs to be supplemented with other characterization methods and models (e.g., chemical and micromechanical) in order to comprehensively address all of the surface-related characteristics important in environmental and other aqueous processes.

## Competing interests

The authors declare that they have no competing interests.

## Authors’ contributions

JRD prepared *Shewanella* cells, carried out EPM experiments and helped to draft the manuscript. YF carried out soft particle modeling and model optimization, interpreted the results and drafted the manuscript. All authors read and approved the final manuscript.

## References

[B1] Pang CM Biofilm formation characteristics of bacterial isolates retrieved from a reverse osmosis membrane Environ Sci Technol 2005 39 19 7541 7550 10.1021/es050170h 16245826

[B2] Bolster CH Haznedaroglu BZ Walker SL Diversity in cell properties and transport behavior among 12 different environmental Escherichia coli isolates J Environ Qual 2009 38 2 465 472 10.2134/jeq2008.0137 19202016

[B3] Flemming HC Wingender J Relevance of microbial extracellular polymeric substances (EPSs) - Part II: technical aspects Water Sci Technol 2001 43 6 9 16 11381977

[B4] Kenward PA Fowle DA Yee N Microbial selenate sorption and reduction in nutrient limited systems Environ Sci Technol 2006 40 12 3782 3786 10.1021/es052210n 16830542

[B5] Gerbersdorf SU Microbial stabilization of riverine sediments by extracellular polymeric substances Geobiology 2008 6 1 57 69 18380886 10.1111/j.1472-4669.2007.00120.x

[B6] Perkins RG Extracellular polymeric substances: quantification and use in erosion experiments Cont Shelf Res 2004 24 15 1623 1635 10.1016/j.csr.2004.06.001

[B7] Spears BM Microalgal sediment biostabilisation along a salinity gradient in the Eden Estuary, Scotland: unravelling a paradox Mar Freshw Res 2008 59 4 313 321 10.1071/MF07164

[B8] Decho AW Microbial exopolymer secretions in ocean environments - their role(s) in food webs and marine processes Oceanogr Mar Biol 1990 28 73 153

[B9] Freeman C Lock MA The biofilm polysaccharide matrix - a buffer against changing organic substrate supply Limnol Oceanogr 1995 40 2 273 278 10.4319/lo.1995.40.2.0273

[B10] Molin S Tolker-Nielsen T Gene transfer occurs with enhanced efficiency in biofilms and induces enhanced stabilisation of the biofilm structure Curr Opin Biotechnol 2003 14 3 255 261 10.1016/S0958-1669(03)00036-3 12849777

[B11] van Duyl FC Tidal coupling between carbohydrate concentrations and bacterial activities in diatom-inhabited intertidal mudflats Mar Ecol Prog Ser 1999 191 19 32

[B12] Hofmann T Dynamics and compositional changes in extracellular carbohydrates in estuarine sediments during degradation Mar Ecol Prog Ser 2009 379 45 58

[B13] Tan X-l Characterization of particle size and settling velocity of cohesive sediments affected by a neutral exopolymer Int J Sediment Res 2012 27 4 473 485 10.1016/S1001-6279(13)60006-2

[B14] Kim J Role of chitin in montmorillonite fabric: transmission electron microscope observations Clays Clay Miner 2012 60 1 89 98 10.1346/CCMN.2012.0600108

[B15] Stumm W Morgan JJ Aquatic chemistry: chemical equilibria and rates in natural waters 1996 New York: Wiley

[B16] Derjaguin B Landau L Theory of the stability of strongly charged lyophobic sols and of the adhesion of strongly charged particles in solutions of electrolytes Acta Physico Chemica URSS 1941 14 633

[B17] Verwey EJW Overbeek JTG Theory of the stability of lyophobic colloids 1948 Amsterdam: Elsevier

[B18] Furukawa Y Aggregation of montmorillonite and organic matter in aqueous media containing artificial seawater Geochem Trans 2009 10 10.1186/1467-4866-10-2 PMC2654465 19166595

[B19] Magal E Colloid transport in porous media: impact of hyper-saline solutions Water Res 2011 45 11 3521 3532 10.1016/j.watres.2011.04.021 21550095

[B20] Phenrat T Aggregation and sedimentation of aqueous nanoscale zerovalent iron dispersions Environ Sci Technol 2007 41 1 284 290 10.1021/es061349a 17265960

[B21] Duval JFL Gaboriaud F Progress in electrohydrodynamics of soft microbial particle interphases Curr Opin Colloid Interface Sci 2010 15 3 184 195 10.1016/j.cocis.2009.12.002

[B22] Ohshima H Electrokinetics of soft particles Colloid Polym Sci 2007 285 13 1411 1421 10.1007/s00396-007-1740-7

[B23] Furukawa Y Watkins JL Effect of organic matter on the flocculation of colloidal montmorillonite: a modeling approach J Coast Res 2012 10.2112/jcoastres-d-11-00128.1

[B24] Ohshima H Electrophoretic mobility of soft particles J Colloid Interface Sci 1994 163 2 474 483 10.1006/jcis.1994.1126 10767166

[B25] Dague E Probing surface structures of *Shewanella* spp. by microelectrophoresis Biophys J 2006 90 7 2612 2621 10.1529/biophysj.105.068205 16415062 PMC1403168

[B26] Neal AL Terminal electron acceptors influence the quantity and chemical composition of capsular exopolymers produced by anaerobically growing Shewanella spp Biomacromolecules 2007 8 1 166 174 10.1021/bm060826e 17206803

[B27] de Kerchove AJ Elimelech M Relevance of electrokinetic theory for “soft” particles to bacterial cells: Implications for bacterial adhesion Langmuir 2005 21 14 6462 6472 10.1021/la047049t 15982054

[B28] De Kerchove AJ Weronski P Elimelech M Adhesion of nonmotile *Pseudomonas aeruginosa* on “soft” polyelectrolyte layer in a radial stagnation point flow system: Measurements and model predictions Langmuir 2007 23 24 12301 12308 10.1021/la701936x 17941654

[B29] Gaboriaud F Coupled electrostatic, hydrodynamic, and mechanical properties of bacterial interfaces in aqueous media Langmuir 2008 24 19 10988 10995 10.1021/la800258n 18512877

[B30] Dale JR Wade R DiChristina TJ A conserved histidine in cytochrome c maturation permease CcmB of *Shewanella putrefaciens* is required for anaerobic growth below a threshold standard redox potential J Bacteriol 2007 189 3 1036 1043 10.1128/JB.01249-06 17142390 PMC1797334

[B31] Ruebush SS Brantley SL Tien M Reduction of soluble and insoluble iron forms by membrane fractions of *Shewanella oneidensis* grown under aerobic and anaerobic conditions Appl Environ Microbiol 2006 72 4 2925 2935 10.1128/AEM.72.4.2925-2935.2006 16597999 PMC1449039

[B32] O’Reilly SE Furukawa Y Newell S Dissolution and microbial Fe(III) reduction of nontronite (NAu-1) Chem Geol 2006 235 1–2 1 11

[B33] Fredrickson JK Reduction of U(VI) in goethite (alpha-FeOOH) suspensions by a dissimilatory metal-reducing bacterium Geochimica Et Cosmochimica Acta 2000 64 18 3085 3098 10.1016/S0016-7037(00)00397-5

[B34] Elzinga EJ ATR-FTIR spectroscopy study of the influence of pH and contact time on the adhesion of *Shewanella* putrefaciens bacterial cells to the surface of hematite Environ Sci Technol 2012 46 23 12848 12855 10.1021/es303318y 23136883

[B35] Gaboriaud F Multiscale dynamics of the cell envelope of Shewanella putrefaciens as a response to pH change Colloids Surf B Biointerfaces 2006 52 2 108 116 10.1016/j.colsurfb.2006.04.017 17120322

[B36] Omoike A Chorover J Spectroscopic study of extracellular polymeric substances from *Bacillus* subtilis: aqueous chemistry and adsorption effects Biomacromolecules 2004 5 4 1219 1230 10.1021/bm034461z 15244434

[B37] Zachara JM Bacterial reduction of crystalline Fe3+ oxides in single phase suspensions and subsurface materials Am Mineral 1998 83 11–12 1426 1443

[B38] Kieft TL Dissimilatory reduction of Fe(III) and other electron acceptors by a *Thermus* isolate Appl Environ Microbiol 1999 65 3 1214 1221 10049886 10.1128/aem.65.3.1214-1221.1999 PMC91167

[B39] Tombacz E Surface modification of clay minerals by organic polyions Colloids and Surfaces a-Physicochemical and Engineering Aspects 1998 141 3 379 384 10.1016/S0927-7757(98)00241-6

[B40] Tourney J Ngwenya BT The effect of ionic strength on the electrophoretic mobility and protonation constants of an EPS-producing bacterial strain J Colloid Interface Sci 2010 348 2 348 354 10.1016/j.jcis.2010.04.082 20546767

[B41] Ohshima H Electrical phenomena of soft particles. A soft step function model J Phys Chem A 2012 116 25 6473 6480 10.1021/jp212002e 22224436

[B42] Abboud R Low-temperature growth of *Shewanella oneidensis* MR-1 Appl Environ Microbiol 2005 71 2 811 816 10.1128/AEM.71.2.811-816.2005 15691935 PMC546687

[B43] Phoenix VR Characterization and implications of the cell surface reactivity of *Calothrix* sp strain KC97 Appl Environ Microbiol 2002 68 10 4827 4834 10.1128/AEM.68.10.4827-4834.2002 12324327 PMC126417

[B44] Tsuneda S Influence of extracellular polymers on electrokinetic properties of heterotrophic bacterial cells examined by soft particle electrophoresis theory Colloids Surf B Biointerfaces 2003 29 2–3 181 188

[B45] Korenevsky AA Characterization of the lipopolysaccharides and capsules of *Shewanella* spp Appl Environ Microbiol 2002 68 9 4653 4657 10.1128/AEM.68.9.4653-4657.2002 12200327 PMC124090

[B46] Claessens J Behrends T Van Cappellen P What do acid–base titrations of live bacteria tell us? A preliminary assessment Aquat Sci 2004 66 1 19 26 10.1007/s00027-003-0687-0

